# Intervention Effect of Group Sensory Integration Training on Social Responsiveness and N170 Event-Related Potential of Children with Autism

**DOI:** 10.3390/bs14030202

**Published:** 2024-03-02

**Authors:** Deming Shu, Gongliang Zhang, Chang Xue, Qiqi Lai, Yueyao He, Yifei Feng, Jianxin Zhang, Fengqin Jia, Dianzhi Liu

**Affiliations:** 1 School of Education, Soochow University, Suzhou 215123, China; shudm@suda.edu.cn (D.S.); glenn@suda.edu.cn (G.Z.); 2 The Autism Research Center, Soochow University, Suzhou 215123, China; 3 School of Sciences, Academy of Future Education, School of Mathematics and Physics, Xi’an Jiaotong-Liverpool University, Suzhou 215123, China; chang.xue21@student.xjtlu.edu.cn (C.X.); qiqi.lai22@student.xjtlu.edu.cn (Q.L.); yueyao.he20@student.xjtlu.edu.cn (Y.H.); yifei.feng21@student.xjtlu.edu.cn (Y.F.); 4 School of Education, Jiangnan University, Wuxi 214122, China; 8201807147@jiangnan.edu.cn; 5 School of Education, Suzhou University of Science and Technology, Suzhou 215009, China

**Keywords:** sensory integration training, autism spectrum disorder, children, event-related potential, N170

## Abstract

The objective of this study was to examine the intervention effect of group sensory integration training on social responsiveness, and the latency and amplitude of N170 event-related potential of children with autism. The social responsiveness scale was employed to assess alterations in the social response of individuals with ASD before and after training, while event-related potentials were utilized to measure changes in N170 latency and amplitude. This study revealed that group sensory integration training can significantly enhance social responsiveness in children diagnosed with ASD. Children with ASD exhibit atypical N170 responses to faces in the right parietal region. The latency of N170 changes may serve as a valuable indicator for assessing the effectiveness of an intervention or diagnosing ASD.

## 1. Introduction

Autism spectrum disorder (ASD) is a pervasive neurodevelopmental disorder that affects individuals worldwide [1,2]. One of the core symptoms of ASD is impaired social communication, which can include difficulties with verbal and nonverbal communication and challenges with social interactions and relationships [3,4]. Impaired face processing is a key feature of social communication deficits in ASD and has been linked to dysfunctions in the neural systems underlying social cognition [5,6].

Research has shown that individuals with ASD exhibit differences in brain function compared with typically developing (TD) individuals [7,8,9,10]. One area of interest in this field is the study of event-related potentials (ERPs). Notably, the N170 is an ERP component and a well-established marker of face processing that has been found to be abnormal in individuals with ASD [11,12,13,14,15]. Accordingly, the N170 component has shown promise as a potential biomarker for assessing face-processing abilities in individuals with ASD [16].

Although some studies have reported changes in ERPs related to sensory processing after training in children with ASD, few have investigated the effects of sensory integration therapy on ERPs specifically related to face processing. For instance, studies have shown that children with sensory processing difficulties may exhibit atypical ERP responses compared to TD children [17]. However, the direct relationship between sensory integration training and ERP changes remains unclear, suggesting that further investigation in this area is warranted.

Impaired facial recognition is an early feature of ASD thought to be associated with social deficits [15,16]. Compared to TD children, children with ASD demonstrate specific impairments in face-processing abilities, which may lead to their experiencing challenges with social interactions and understanding others’ emotions [6,9]. Specifically, indicators of face-processing difficulties in individuals with ASD include lack of eye contact, atypical gaze patterns, and reduced interest in faces [18]. These impairments in face recognition and, relatedly, social communication skills significantly affect the daily functioning and social integration of individuals with ASD [19,20]. Although various interventions have been developed to address these difficulties, more objective measures are required to evaluate treatment outcomes [21].

In response to the need for such interventions to support children diagnosed with ASD, especially in terms of social functioning [22], this study explored the effectiveness of an intervention based on sensory integration theory. Specifically, based on the concept of integrating sensory information with oriented behaviors, sensory integration theory integrates the neurological processing of sensory information, which has been proven to improve autism-related symptoms [23]. This study explored whether conducting group sensory integration training for six weeks with children with ASD aged 5–12 years changed the N170. Specifically, we tested whether the training decreased the N170 latency elicited by faces in the right parietal regions of the participants. Ultimately, this study aimed to investigate the effectiveness of sensory integration training on face-processing skills in children with ASD and determine the utility of the N170 as an indicator for evaluating treatment effects. Insights into the neurophysiological changes associated with interventions can enhance the development of targeted and evidence-based interventions for individuals with ASD to improve their facial recognition and social communication abilities.

## 2. Methods

### 2.1. Participants

Sixteen children with ASD participated in this study. Data obtained from four children with ASD were excluded from the analysis because of difficulties in acquiring EEG data (e.g., lack of patience to complete the whole task or refusal to wear an electrode cap). The final analysis involved 12 children with ASD (11 boys and 1 girl; age range: 5–10 years; mean age: 7.33 ± 1.49 years) and 12 TD children (9 boys and 3 girls; age range: 5–10 years; mean age: 8.42 ± 1.99 years). Statistical testing revealed no significant differences in gender and age between the participants with ASD and the TD participants (gender, *χ*^2^ = 1.20, *p* = 0.27; age, *t*(22) = 1.51, *p* = 0.15). All participants with ASD were diagnosed by professional pediatric clinicians with expertise in child psychiatry and met the diagnostic criteria for autism in the DSM-V [24], which was used to confirm the diagnosis of children with ASD. Before the experiment, the participants’ caregivers provided written informed consent in accordance with the 1964 Declaration of Helsinki. This study was approved by the Ethics Committee of School of Education, Soochow University.

### 2.2. Group Sensory Integration Training

Children with ASD received 20 sessions of sensory integration training over a six-week period. Each session involved approximately one hour of group training for sensory integration. The intervention training was conducted with a one-day interval to ensure that the children were adequately rested and had sufficient time for consolidation. Intervention was conducted through group-based games, with each group consisting of 5–7 participants, and followed a sequential order for mutual imitation. Each child was required to be accompanied by a primary caregiver, who assumed the responsibility of guiding the child’s attention. To address the issue of high heterogeneity among children, varying degrees of assistance were provided to ensure each child’s successful completion of the task.

The training consisted of four stages: basic motor skill familiarity (five sessions), initial combination of basic motor skills and motor skills (five sessions), joint motor skill transfer (five sessions), and comprehensive application of motor skills (five sessions).

In the first stage, each session comprised four distinct parts: Part 1 for conducting warm-up exercises; Part 2 involved ice-breaker games; Part 3 encompassed fundamental motor skills training, such as climbing, walking, running, and jumping movements, hands-and-knees crawling, beam walking, backwards running, and open and closed jumping training to stimulate the children’s tactile, vestibular, and proprioceptive senses while also enhancing their recognition of sensory stimuli and understanding of fundamental motor skills related to displacement; and Part 4 involved performing body stretching exercises, where imitation-based learning was primarily employed by the facilitators, volunteers, and children.

In the second stage, each session comprised four distinct parts: Part 1 for conducting warm-up exercises; Part 2 reinforced the motor skills acquired in the first stage and fostered peer collaboration skills; and Part 3 was interactive games involving object manipulation movements, such as ball throwing, catching, and shooting; Part 4 involved performing body stretching exercises, to enhance the children’s proprioceptive development through object manipulation movements.

In the third stage, each session comprised five distinct parts, encompassing the four parts of the second stage, as well as a part dedicated to group games. This stage involved facilitating the transfer of the children’s skill application across diverse scenarios or through the utilization of alternative equipment, thereby promoting the effective integration of sensory stimuli in children.

In the fourth stage, each session comprised five distinct parts, similar to the third stage, but with a focus on comprehensive training of abilities that enhanced displacement and object manipulation movements. Additionally, it emphasized group-based learning and play to facilitate the children’s spontaneous and appropriate responses to sensory stimuli. The activities were structured in an advanced progression from simple to complex, primarily highlighting the correlation between fundamental motor skills and sensory integration capabilities. Furthermore, they encompassed various visual, auditory, tactile, proprioceptive, and vestibular sensory stimuli.

### 2.3. Social Responsiveness Scale

The social responsiveness scale (SRS) is a brief assessment tool for measuring autistic traits and quantitatively assessing ASD in children and adolescents [25]. It is sensitive enough to detect even subtle symptoms and specific enough to differentiate between clinical groups, both within the autism spectrum and between ASD and other disorders. All items are rated on a 4-point Likert scale anchored by “not true” and “almost always true”, where higher scores indicate greater dysfunction. The five subscales are social awareness, social cognition, social communication, social motivation, and restricted interests and repetitive behaviors. The SRS scores of individuals with ASD were assessed both pre and post intervention in this study. All SRS scales were completed by parents of children diagnosed with ASD; all parents underwent comprehensive training and on-site guidance provided by professionals to ensure the accuracy and reliability of data collection.

### 2.4. Event-Related Potentials

Children with ASD were required to complete the experimental tasks on a computer before and after the training while recording the EEG and analyzing the component characteristics of ERPs elicited by the experimental tasks offline. Twelve TD children (the control group) completed the same task.

### 2.5. Experiment Design and Procedure

The stimuli consisted of 300 distinct grayscale digital images of affective faces (150 male and 150 female faces drawn from the Chinese Affective Face Picture System, CAFPS; ref. [26]), 50 non-face stimuli, and 50 target pictures (pictures of the moon). We conducted a total of 400 trials.

**Figure 1:**
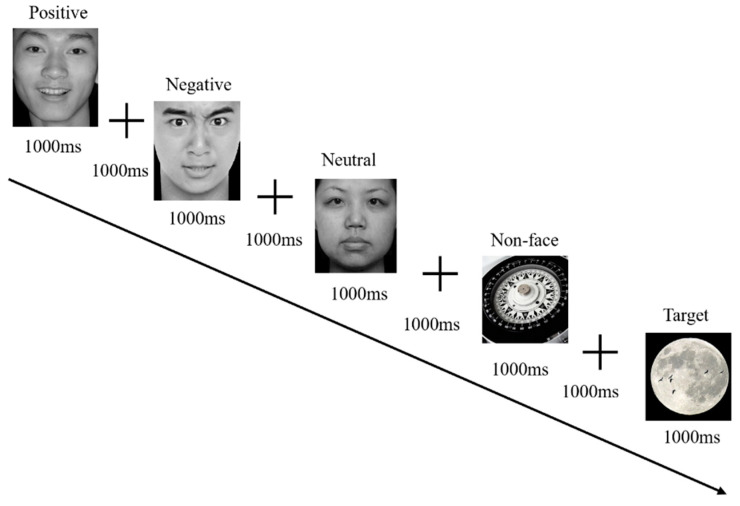
Flow chart of event-related potential experimental stimuli.

All stimuli were presented in frontal view and in a standardized viewing size (stimulus: 5.5° × 6.2°; cross-fixation: 1.3° × 1.3°) on a uniform black background. Stimuli were presented on a 27-inch color monitor (144 Hz, 2560 × 1440 resolution) with E-Prime 2.0 software at a viewing distance of 65 cm in a sound-attenuated room with low ambient illumination. The cross-fixation was presented for 1000 ms. The stimulus was presented for 1000 ms. A rest was scheduled in the middle of the experiment. Figure 1 details the process. The whole process lasted 13.3 min. EEG data were recorded continuously at 500 Hz using an online 0.016 Hz high-pass filter. There were 32 channels. Impedances were kept below 10 kΩ. The cross-fixation disappeared simultaneously with the presentation of a randomly selected face and an image of something other than a face (the non-face image) for 1000 ms to monitor attention. Participants were instructed to press a button upon detecting randomly interspersed target stimuli (50 times). Target trials were excluded from the analysis.

## 3. Results

### 3.1. SRS Scores Exhibit Alterations Pre and Post Intervention

The intervention led to a significant improvement in SRS scores of individuals with ASD. Table 1 shows the changes in SRS scores pre and post intervention. The paired sample *t*-test revealed a statistically significant difference in the total SRS scores before and after the intervention (*t*(11) = 2.55, *p* = 0.03, *d* = 0.73). The scores on the social awareness (AWR), social communication (COMM), and restricted interests and repetitive behaviors (RRB) subscales significantly decreased following the intervention (AWR: *t*(11) = 3.40, *p* < 0.01, *d* = 0.99; COMM: *t*(11) = 2.21, *p* = 0.049, *d* = 0.63; RRB: *t*(11) = 2.90, *p* = 0.01, *d* = 0.84)). These findings indicated an improvement in the social functioning of children with ASD following the intervention.

### 3.2. EEG Processing and Statistical Analysis

The data were processed using a 0.016 Hz online filter and a 30 Hz low-pass digital filter. A 100 ms pre-stimulus baseline period was subtracted from each trial before averaging to correct for baseline shifts. The segmentation epoch was 100 ms before and 500 ms after stimulus onset. Artifact detection settings were set to 100 μv for bad channels. Time windows for ERP analysis were chosen by visual inspection of grand-averaged data and confirmed for individual averages.

#### 3.2.1. Latency of the N170

**Figure 2:**
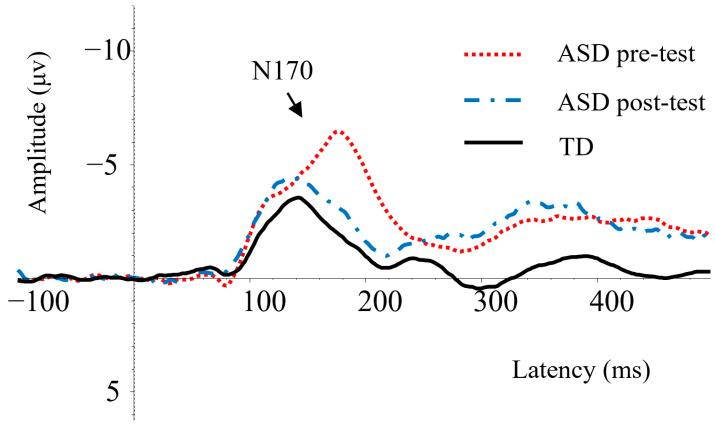
Grand averages of ERP waveforms in the right parietal region.

**Figure 3:**
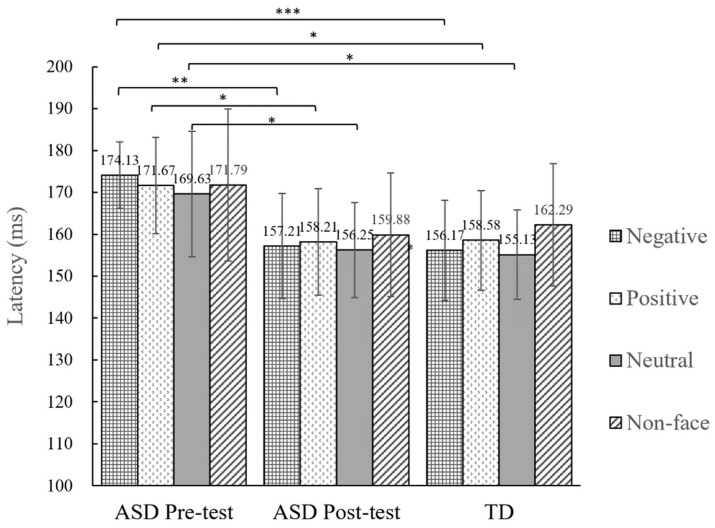
Comparison of N170 latency elicited by different types of stimuli in the right parietal region. * *p* < 0.05, ** *p* < 0.01, *** *p* < 0.001.

The resultant time windows were extended from 140 to 200 ms for the N170. The selected time windows are consistent with the N170 time windows described in prior studies [16,27]. Previous studies have revealed that the distribution of the N170 mainly appears in the occipitotemporal region and protrudes in the right hemisphere, demonstrating a negative deflection potential that peaks at 170 ms [28,29,30]. This study employed 32 electrode caps and focused on the right parietal sites—comprising electrodes CP2, CP6, Pz, and P4—as the region of interest (ROI). The grand-averaged ERP waveforms of the right parietal region are shown in Figure 2. The average values of N170 latency elicited pre and post intervention in children with ASD and TD children under different conditions are shown in Figure 3.

The data were analyzed using a repeated-measures analysis of variance (ANOVA) with a 2 (test time: pre-test, post-test) × 4 (stimulus type: negative emotional faces, positive emotional faces, neutral emotional faces, and non-face) design in SPSS 22.0. The results revealed significant test time main effects (*F*(1,11) = 10.24, *p* < 0.01, $\eta_{p}^{2}$ = 0.48). The latency of the N170 in the post-test was significantly shorter than that in the pre-test. The statistical analysis found that the stimulus types did not have any statistically significant main effects (*F*(3,33) = 0.56, *p* = 0.65, $\eta_{p}^{2}$ = 0.05). Additionally, the statistical analysis did not reveal any significant findings when examining the interactions between test time and stimulus types (*F*(3,33) = 0.32, *p* = 0.81, $\eta_{p}^{2}$ = 0.03). The differences in the pre- and post-test results for different types of stimuli were compared using paired sample *t*-tests, revealing a significant difference in the latency of the N170 elicited by all types of emotional faces in all children with ASD (negative: *t*(11) = 4.41, *p* < 0.01, *d* = 1.27; positive: *t*(11) = 2.74, *p* = 0.02, *d* = 0.79; neutral: *t*(11) = 2.72, *p* = 0.02, *d* = 0.78). The latency of the N170 was significantly shortened in the post-test. However, there was no statistically significant difference in the latency of the N170 elicited by non-face stimuli between the pre- and post-tests (non-face, *t*(11) = 1.61, *p* = 0.14, *d* = 0.46)).

The pre- and post-tests of the ASD group were compared with those of the TD group using independent-sample *t*-tests. The pre-test for ASD revealed a significant difference in the N170 latency elicited by faces compared with that observed in TD children (negative, *t*(22) = 4.34, *p* < 0.001, *d* = 1.77; positive, *t*(22) = 2.74, *p* = 0.01, *d* = 1.12; neutral, *t*(22) = 2.73, *p* = 0.01, *d* = 1.12). Notably, the N170 latency elicited by the pre-test faces in the ASD group was significantly prolonged compared with that in the TD group. The N170 latency elicited by the pre-test non-faces in the ASD group was not significantly different from that in the TD group (*t*(22) = 1.41, *p* = 0.17, *d* = 0.58). No significant differences in N170 latency were observed between the post-test results for individuals with ASD and TD across all types of stimuli.

#### 3.2.2. Peak Amplitude of the N170

**Figure 4:**
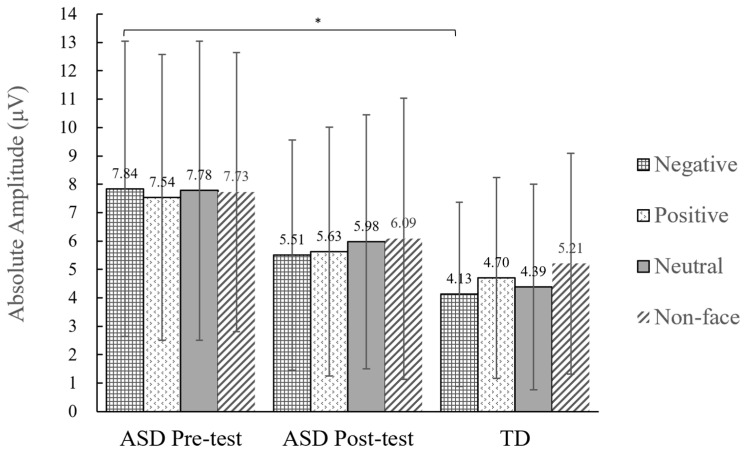
Comparison of N170 peak amplitude elicited by different types of stimuli in the right parietal region. * *p* < 0.05.

The peak amplitude of the N170 was averaged across the electrode group for each participant and exported to SPSS 22.0. A comparison of the average values is shown in Figure 4. The N170 peak amplitude data were analyzed using a repeated-measures ANOVA with a 2 (test time: pre-test, post-test) × 4 (stimulus type: negative emotional faces, positive emotional faces, neutral emotional faces, and non-face) design. The results revealed no significant main effects for the pre-test and post-test conditions (*F*(1,11) = 1.15, *p* = 0.31, $\eta_{p}^{2}$ = 0.10). The amplitudes of the N170 in the pre- and post-test were not significantly different. The statistical analysis reveals that the stimulus types did not have any statistically significant main effects on N170 amplitude (*F*(3,33) = 0.28, *p* = 0.84, $\eta_{p}^{2}$ = 0.03). Further, the statistical analysis did not reveal any significant findings regarding the interactions between test time and stimulus types (*F*(3,33) = 0.18, *p* = 0.91, $\eta_{p}^{2}$ = 0.02). The differences in the pre- and post-test results for different types of stimuli were compared using paired sample *t*-tests, which revealed no significant differences in amplitude. The difference in the N170 elicited by negative facial stimuli between the TD and ASD groups was found to be statistically significant (*t*(22) = −2.10, *p* = 0.048, *d* = 0.86). The amplitude of the pre-test in the ASD group was larger than that in the TD group. However, there was no statistically significant difference in N170 amplitude elicited by other stimuli between the ASD and TD groups.

## 4. Discussion

### 4.1. Effects of the Group Sensory Integration Training

Among children with ASD, there is a significantly high prevalence of sensory integration disorders, which greatly impede the psychological and behavioral development of these individuals. This in turn hinders their acquisition of essential life skills and gives rise to various behavioral and social adjustment barriers. Sensory integration training, which leverages the neuroplasticity observed during the developmental stages of children with autism, employs interactive games, artistic activities, and scientific movements to facilitate novel sensory integration and motor experiences that can effectively rewire existing neural connections and pathways within the brain. Consequently, SIT has been shown to enhance language abilities, social interaction capabilities, sensory perception skills, and overall behavior among children diagnosed with ASD. The optimal age for intervention utilizing sensory integration training ranges from 2 to 11 years. This study found that the SRS scores of children with ASD significantly improved after they participated in 20 sessions of a group sensory integration training over 6 weeks. Specifically, the post-test total SRS score (86.83 ± 12.64) was significantly lower than the pre-test score (94.25 ± 14.49), indicating that the social functioning of children with ASD substantially improved following the group sensory integration training. The post-test scores for the social awareness, social communication, and restricted interests and repetitive behaviors subscales were significantly lower than the pre-test scores, indicating significant improvements in these areas following the group sensory integration training for individuals with ASD.

Research has already suggested that the implementation of social skills training interventions significantly improves the social skills of children with ASD [31]. In particular, sensory integration therapy—which is based on the theory that the brain can integrate sensory information from different areas of the body to improve information-processing abilities [32]—is a widely used intervention in children with ASD. Sensory integration therapy typically involves activities that stimulate the senses, such as swinging, bouncing, and brushing, and has been shown to effectively improve social behavior and psychological states in children with ASD [33,34]. However, the effects of sensory integration therapy on facial recognition impairments in children with ASD are poorly understood and not all sensory integration training interventions for children with ASD have demonstrated consistent positive outcomes [35].

In light of these findings, the positive impact of sensory integration training observed in this study may be attributed to the use of group training. Social dysfunction is a fundamental challenge for children with ASD, who must accordingly develop intricate abilities to function in social situations. In contrast to traditional one-on-one sensory integration training, our group intervention incorporated extensive social interactions and advanced sensory integration techniques—the group setting compels peers to engage in increased interactions, learning, and imitation. Children who engaged in group sensory integration training games fostered their intrinsic interest and observational learning. Throughout the training, parents actively participated and accompanied their children while also acquiring relevant intervention methods to facilitate continued practice at home. By facilitating natural and sophisticated social scenarios in which children with ASD can develop social skills, group sensory integration training offers significant potential for enhancing the social competence of individuals with ASD. In comparison to the traditional one-on-one approach, group sensory integration training effectively incorporates social interaction, observational learning, joint attention, and sensory integration training. This comprehensive approach enhances the overall social and sensory integration abilities of children with ASD. Our results demonstrating changes in the SRS scale and N170 latency show that we may be able to improve both motor and cognitive nervous systems in ASD children by utilizing physical movement and sensory integration techniques.

### 4.2. N170 and Face Processing in Children with ASD

We employed an ERP paradigm to investigate differences in N170 latency and amplitude among children with ASD and their TD peers before and after the group sensory integration training.

#### 4.2.1. Changes in N170 Latency Pre and Post Intervention

We found that the N170 latency elicited by faces within the right parietal region was significantly reduced among children with ASD after the intervention. Notably, a significant discrepancy in the N170 latency between individuals with ASD and TD was observed prior to the implementation of the intervention; however, this discrepancy was statistically insignificant after the intervention. Importantly, no such alteration was detected in non-face-elicited N170 latency.

As established above, the N170 is an ERP component that can be triggered by faces and is believed to reflect the structural encoding of faces [36]. A meta-analysis proved that the N170 is sensitive to facial expressions [13]. Multiple studies have shown that longer latency periods are associated with face-processing difficulties in ASD [30,37,38,39]. Previous ERP studies investigating face processing have demonstrated that individuals with ASD across different age groups, including children, adolescents, and adults, exhibit delayed N170 latencies compared to TD individuals [36,40,41]. After 16 weeks of pivotal response treatment (PRT), one study revealed significant reductions in N170 latency to faces, whereas no changes in N170 latency were observed in the waitlist-control condition [38].

Currently, there are no objective biomarkers of ASD that are useful for clinical care and research. However, an abnormal N170 index reflects nonspecific facial affective processing disorders, and thus serves as a biomarker for face recognition [42]. The results of this study emphasize the specificity of N170 latency in children diagnosed with ASD and underscore its potential utility as a reliable indicator for assessing intervention outcomes. In particular, this study evidences that N170 latency can reflect the effects of behavioral therapy.

#### 4.2.2. Pre-Test N170 Amplitude Is Significantly Larger among Children with ASD Than in TD Children

Compared with children with TD, children with ASD exhibited significantly larger N170 amplitudes in the right parietal region when presented with negative facial stimuli prior to the intervention. No significant differences were observed in responses to other faces or non-face stimuli. Previous studies have found that if the face is inverted, there are some obstacles to face recognition; in these cases, the amplitude of the N170 is larger and the latency is longer [30]. We found that the N170 amplitude elicited by positive and neutral emotional faces in the pre-test did not significantly differ between ASD and TD children. However, we only observed a significant difference for negative emotional faces; this finding suggests that negative emotional faces pose greater difficulties for children with ASD. Notably, a meta-analysis revealed delayed N170 latency during face recognition tasks in individuals with ASD without any discernible differences in amplitude [36]. This finding can be attributed to the fact that previous studies have not specifically examined the N170 responses elicited by negative emotional faces. In contrast, this study comprehensively analyzed N170 amplitudes based on various types of emotional faces for a more rigorous investigation.

Our observations above imply that the N170 amplitude elicited by negative emotional faces may demonstrate enhanced sensitivity in evaluating intervention effects or serving as diagnostic indicators for ASD, relative to other emotional faces.

## 5. Conclusions

This study found that group sensory integration training significantly enhanced social responsiveness among children with ASD. Notably, we studied this effect in light of changes in the N170 among children with ASD compared to that of their TD peers. As established above, the N170 elicited by face processing differs between children with ASD and TD children. Specifically, in studying the right parietal regions of children with ASD in comparison to those of TD children, we found that the latency of the N170 elicited by faces was significantly delayed and the amplitude of the N170 induced by negative emotional faces was significantly increased in children with ASD. These findings suggest that the latency of the N170 in the right parietal region may serve as a potential diagnostic indicator of ASD. Moreover, this study also evidenced the neural plasticity underlying facial processing in children with ASD: following the group sensory integration training, N170 latency significantly decreased during face processing but not in response to a non-face stimuli. These results suggest that facial N170 latency may serve as an objective measure to evaluate the efficacy of interventions.

## 6. Limitations

It is important to note that this study had a relatively small sample size, consisting of only 12 children who were diagnosed with ASD. This limitation arises from the inherent challenges associated with conducting experiments involving children with autism, including those related to their young age and symptoms. Given the limited sample, caution should be exercised when generalizing these outcomes to a broader population of children with ASD. Further studies with larger sample sizes are required to validate and expand upon these findings. Another limitation is related to the need for further investigation into whether the recovery of certain neurological functions following an intervention is transient or enduring. Therefore, future studies should involve larger samples and extended observations of the rehabilitative impact of the intervention on neurological function in individuals with autism to investigate the feasibility and durability of neurobiological changes.

## Figures and Tables

**Table 1:** Comparison of SRS scores before and after the intervention.

	Pre-Test	Post-Test	T	d
	M (SD)	M (SD)		
SRS Total	94.25 (14.49)	86.83 (12.64)	2.55 *	0.73
AWR	13.08 (2.61)	11.67 (2.74)	3.40 **	0.99
COG	20.25 (3.22)	18.33 (3.11)	1.65	0.48
COMM	30.83 (5.59)	28.75 (4.69)	2.21 *	0.63
MOT	13.5 (4.62)	13.67 (3.50)	−0.19	0.06
RRB	16.58 (4.08)	14.42 (3.75)	2.90 *	0.84

Note: SRS = social responsiveness scale, AWR = awareness, COG = cognition, MOT = motivation, COMM = communication, RRB = restricted interests and repetitive behaviors, M = mean, SD = standard deviation, d = effect size, * *p* < 0.05, ** *p* < 0.01.

## Data Availability

Data will be made available on request.
